# Online Siamese Network for Visual Object Tracking

**DOI:** 10.3390/s19081858

**Published:** 2019-04-18

**Authors:** Shuo Chang, Wei Li, Yifan Zhang, Zhiyong Feng

**Affiliations:** 1 School of Information and Communication Engineering, Beijing University of Posts and Telecommunications, Beijing 100876, China; zhangyf@bupt.edu.cn (Y.Z.); fengzy@bupt.edu.cn (Z.F.); 2 Department of Electrical Engineering, Northern Illinois University, Dekalb, IL 60115, USA

**Keywords:** visual object tracking, Siamese network, improved contrastive loss, Bayesian verification

## Abstract

Offline-trained Siamese networks are not robust to the environmental complication in visual object tracking. Without online learning, the Siamese network cannot learn from instance domain knowledge and adapt to appearance changes of targets. In this paper, a new lightweight Siamese network is proposed for feature extraction. To cope with the dynamics of targets and backgrounds, the weight in the proposed Siamese network is updated in an online manner during the tracking process. In order to enhance the discrimination capability, the cross-entropy loss is integrated into the contrastive loss. Inspired by the face verification algorithm DeepID2, the Bayesian verification model is applied for candidate selection. In general, visual object tracking can benefit from face verification algorithms. Numerical results suggest that the newly developed algorithm achieves comparable performance in public benchmarks.

## 1. Introduction

As a fundamental and challenging task, visual object tracking has a variety of applications, such as smart video surveillance, autopilot, human–computer interaction and video communication [1,2,3]. In general, the goal of visual object tracking is to estimate the position and scale variation of targets in the video sequence, where its initial state is given in the first frame. Object occlusion, scale variation, illumination variation, deformation, background clutter and motion blur are the major challenges for robust visual object tracking.

Recently, convolutional neural networks (CNNs) achieved great success in image classification, semantic segmentation, object detection, and other computer vision problems [4,5,6]. Tracking algorithms [7,8,9,10,11,12,13] with CNNs for feature extraction have attained state-of-the-art results on the visual tracking benchmarks [14]. Compared to hand-crafted feature models [15], CNN-based algorithms could learn a specific feature model with large-scale training data. They are more robust to the deformation of objects.

To exploit the representation capabilities of CNNs, Tao et al. [9] proposed a matching function with the Siamese network to extract feature vectors, which was named the Siamese Instance Search for Tracking (SINT). This new method was trained using the contrastive loss. Similarly, in [7], a fully convolutional Siamese-like tracking algorithm (SiamFC) was proposed, which was supervised by the logistic loss.

The above two algorithms train the Siamese Networks in an offline manner. Ideally, feature vectors of the same target in different frames need to be close to each other. In order to train such a CNN model, a large set of annotated video sequences are needed. However, in practice, the amount of annotated video sequences for tracking is insufficient. For example, only 3964 groups of annotated video sequences are used in the offline training in SiamFC, which are extracted from ImageNet [16]. Without online updating, the offline learned Siamese models are susceptible to appearance changes.

Different from SiamFC and SINT, the algorithms MDNet and SANet trained an offline model and updated part of it in the inference phase, and these two algorithms were supervised by logistic loss. In addition, these two algorithms have made superior performance in online tracking benchmark (OTB). However, the dataset for model training (i.e., in the offline process) in MDNet [10] and SANet [11] are not completely independent with regard to the test dataset, and the training data used in MDNet [10] and SANet [11] is not supported by the visual object tracking (VOT) [17]. At the same time, discriminative correlation filter (DCF) algorithms like ECO [12] and MCPF [13] have been proposed, and they update their tracking models with historical prediction results. Basically, the DCF methods train a regression model to indicate object’s position offset, which is different from MDNet, SANet, and our proposed algorithm.

**Figure 1:**
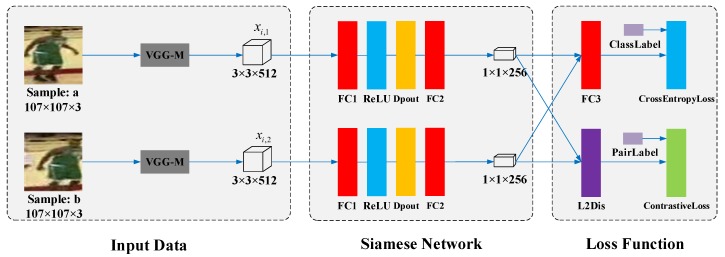
The framework of Siamese Network in OSNV. **Left**: **Input Data** for updating **Siamese network**, which are the outputs of layer conv3 in VGG-M. Our proposed **Siamese network** lies in the middle, and two groups of branches share the same configuration and weights. **Right**: the improved contrastive loss, which is served as the **Loss Function** to propagate gradients for updating the **Siamese network**. Dpout stands for drop-out layer, best viewed in colour.

In this paper, a novel online lightweight Siamese network is proposed for visual tracking (OSNV). In the tracking inference phase, tracking results and sample candidates are collected to maintain an online training set. Unlike SiamFC and SINT, the proposed method updates the feature model with historical prediction results. Because of that, the feature model could learn from domain knowledge and adapt to appearance changes of target. By the way, the input data of the proposed Siamese network is the feature maps of VGG-M [18], not the raw image data. The framework of online Siamese Network is depicted in Figure 1.

In addition, the offline Siamese network trained by the contrastive loss [19] or logistic loss [7] has limited discrimination capability. Furthermore, it is not easy to prepare a dataset to train a Siamese network. If the cross-entropy loss is adopted [20], the feature extraction model may be overfitting due to limited training data. On the other hand, the contrastive loss and logistic loss mainly concentrate on reducing the intra-class variations while the cross-entropy loss leads to increasing the inter-class variations. In conclusion, they are complementary to each other. With these properties, the cross-entropy loss [21] is integrated with the contrastive loss to update the proposed Siamese network, which is helpful to enhance the model’s discrimination.

As for candidate selection, the Bayesian verification model [22] is used. It is widely used as a matching function in the face verification task [22,23]. Instead of identifying a hyperplane to distinguish the object from the background, the Bayesian verification model, trained using expectation maximization (EM), determines a log likelihood ratio to give a similarity score for an image-pair.

The main contributions of this paper are summarized as follows:

- An online Siamese network is proposed. It can learn from the domain knowledge of target and adapt to appearance changes of target;
- An improved contrastive loss integrated with cross-entropy loss is introduced to update the Siamese network;
- The Bayesian verification model is transferred for candidate selection. In addition, we find that the visual object tracking can benefit from face verification algorithms;
- Four ablation experiments are applied to verify the effectiveness of the proposed loss function. The obtained numerical results demonstrate that the newly developed Siamese network outperforms SiamFC [7] and SINT [9], and has made a comparable performance with state-of-the-art trackers.

The rest of this paper is organized as follows. Section 2 reviews the related work. In Section 3, details of proposed Siamese network and the improved loss function are presented. Implementation details about Bayesian verification model are provided in Section 4. The numerical results are provided in Section 5. Finally, Section 6 concludes the whole paper.

## 2. Related Works

### 2.1. Siamese Network for Visual Object Tracking

For the Siamese network, Tao et al. [9] proposed a Siamese model with a region of interest (e.g., RoiPool [24]) layer. It keeps the feature vectors with different regions the same length. Their proposed feature model learns a matching mechanism [9]. The SINT algorithm [9] samples candidates in the coming frame and the most similar one is determined by the learned matching function. However, without online updating, SINT cannot learn from the domain knowledge with specific tracking targets.

Different from SINT, the SiamFC method [7] is supervised by the logistic loss. It has an exemplar CNN branch and a search CNN branch. These two branches share the same weight and configuration. The output feature maps of the exemplar correlates with the search branch to generate a response map. The location of the maximum value within the response map indicates the position offset of target. However, without the online updating, the SiamFC cannot adapt to appearance changes of tracking targets. Similar to SiamFC, Guo [25] proposed a dynamic Siamese network, which is robust to target variation and background clutters. In the work of DCFNet [26], Wang et al. proposed a correlation filter layer, which is inserted in the Siamese network to learn the convolutional features and enlarge the training dataset simultaneously. In addition, the prediction results returned by the SiamFC are penalized by a cosine window in order to achieve a comparable performance. In our proposed algorithm, the confidence of prediction result is similarity score returned by Bayesian verification model without any window tricks.

### 2.2. Online Algorithms for Visual Object Tracking

Online models for visual object tracking mainly have two classes: DCF based and None-DCF based. DCF-based algorithms i.e., CF2 [8], ECO [12], MCPF [13], SRDCF [27], train correlation filters based on feature vectors extracted from pre-trained CNN models. For the None-DCF based algorithms, the representative trackers are MDNet and SANet. They train an offline model with a tracking dataset, where part of the feature model is updated in tracking. However, the dataset for model training (i.e., in the offline process) in MDNet and SANet is not completely independent with regard to the test dataset, which is not supported by the VOT-Challenge [17].

### 2.3. Loss Function for CNNs in Visual Tracking

In [20], Wang et al. proposed an online CNN model, which is supervised by the cross-entropy loss. The cross-entropy loss function aims at finding a hyperplane to distinguish the target from the background and increases the inter-class variations. It is always used in the classification task [4,21]. However, the cross-entropy loss is prone to overfitting due to the limited training data, which makes it not robust to appearance changes of targets. For the offline-trained models, the contrastive loss is used in [9] and the logistic loss is used in [7]. The contrastive loss and logistic loss are mainly used to reduce the intra-class variations, which make them robust to the distractor from similar objects. In this paper, we propose an improved loss function where the cross-entropy loss is integrated with the contrastive loss.

### 2.4. Bayesian Verification Model

Bayesian formulation has been very successful in the face verification task. In [22], Chen et al. proposed a new Joint Bayesian formulation based on the classical Bayesian face recognition method. Instead of modeling the difference of appearance between two images, they set up a joint Gaussian distribution for an image pair. The method yielded an excellent result on the challenging data set of Labeled Face in Wild (LFW) [28] with ”hand-crafted features”. In [23], Sun et al. constructed a DeepID2 CNN model to map pixel values into the high-dimensional feature space, which is a Siamese-like structure as well. They also trained a Bayesian model with input data extracted by the DeepID2 CNN network for face verification. Similar to DeepID2, the Bayesian verification model is implemented for candidates selection.

With Bayesian verification [22], a target is modeled by summing two independent Gaussian variables as

(1) x=μ+ε,

(2) $$\mu ∼N\left(0,S_{\mu }\right),\varepsilon ∼N\left(0,S_{\varepsilon }\right).$$

μ and ε are latent variables, and their covariance matrices are $S_{\mu }$, $S_{\varepsilon }$, respectively. The log likelihood ratio $R(x_{i},x_{j}|H_{I},H_{E})$ for two samples ($x_{i},x_{j}$) is:

(3) $$R\left(x_{i},x_{j}|H_{I},H_{E}\right)=\log\frac{P(x_{i},x_{j}|H_{I})}{P(x_{i},x_{j}|H_{E})},$$

where $P(x_{i},x_{j}|H_{I})$ is the probability that the two samples ($x_{i},x_{j}$) are from the same target. $P(x_{i},x_{j}|H_{I})$ is Gaussian with covariance matrix:

(4) $$\Sigma_{I}=\left[\begin{array}{cc} S_{\mu }+S_{\varepsilon } & S_{\mu }\\ S_{\mu } & S_{\mu }+S_{\varepsilon } \end{array}\right].$$

$P(x_{i},x_{j}|H_{E})$ is the probability that the two samples ($x_{i},x_{j}$) are from different targets. The associated covariance matrix is:

(5) $$\Sigma_{E}=\left[\begin{array}{cc} S_{\mu }+S_{\varepsilon } & 0\\ 0 & S_{\mu }+S_{\varepsilon } \end{array}\right].$$

From Labels (4) and (5), the log likelihood ratio in Label (3) can be expressed as [22], after ignoring constant terms,

(6) $$\begin{array}{cc} R(x_{i},x_{j}|H_{I},H_{E}) & =\log\frac{P(x_{i},x_{j}|H_{I})}{P(x_{i},x_{j}|H_{E})}\\ & =x_{i}^{T}Ax_{i}+x_{j}^{T}Ax_{j}-2x_{i}^{T}Gx_{j}, \end{array}$$

where

(7) $$A={\left(S_{\mu }+S_{\varepsilon }\right)}^{-1}-\left(F+G\right),$$

(8) $$\left[\begin{array}{cc} F+G & G\\ G & F+G \end{array}\right]={\left[\begin{array}{cc} S_{\mu }+S_{\varepsilon } & S_{\mu }\\ S_{\mu } & S_{\mu }+S_{\varepsilon } \end{array}\right]}^{-1}.$$

The parameters $\Theta =\{S_{\mu },S_{\varepsilon }\}$ can be learned by the EM-like algorithm [22].

## 3. Proposed Algorithm

In this section, details of the proposed Siamese network are described. Then, a brief introduction about contrastive loss and cross-entropy loss is given. After that, the improved loss function is presented. Finally, we talk about the implementation of Bayesian Verification model for candidates’ selection.

### 3.1. Siamese Network

The proposed Siamese network for online updating consists of two branches, and they share the same configurations and weight. As shown in Figure 1, each branch of the Siamese network consists of four CNN layers. There are three types of CNNs layers including two fully convolutional layers (FC1 and FC2), a rectified linear unit layer (ReLU) and a Dropout layer (Dpout). The configurations of filters size, stride and pad within the layers FC1 and FC2 are (3×3×512×512, 1, 0) and (1×1×512×256, 1, 0), respectively. The purpose is to gradually reduce the feature dimension and remove redundancy.

**Figure 2:**
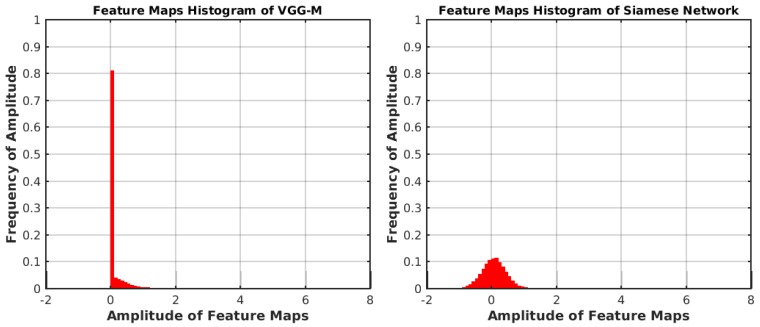
The histograms of feature maps with different feature models. **Left**: Histogram of VGG-M; **Right**: Histogram of our Siamese network.

To illustrate the effect of the proposed Siamese network, we collect the feature maps from conv3 in VGG-M and our proposed Siamese network. These feature maps are obtained by the first frame of 100 video sequences in OTB-2015 [29]. The histograms of the collected feature maps are depicted in Figure 2. The width of the bins is set to be 0.1. As shown in Figure 2, we can see that the amplitude frequency in the range of [0,0.1) is 0.8114. Moreover, in the range of [0,0.1), 94.84% amplitude values are equal to 0. Therefore, the feature maps of conv3 in VGG-M are sparse and there exists redundant information, which are not relative to visual tracking task. With our proposed Siamese network, the histogram of the obtained feature maps is depicted in Figure 2. The frequency of the amplitude value range [0,0.1) is now 0.1119. Moreover, points with zero amplitude are removed. In other words, our proposed Siamese network effectively eliminates redundant information.

Due to the training data scarcity, a lightweight Siamese network with four layers is designed. Because of online updating, the feature model may be overfitting to historical target’s appearance. To address this issue, we add a drop-out layer [30] after the layer ReLU (see Figure 1). We set the drop-out rate as 0.5. For the layer FC3, it has two outputs, corresponding to the target and background. The new layer L2Dis is used to calculate the square of Euclidean distance between two feature vectors by:

(9) $$d_{i}={∥f\left(x_{i,1},w_{0}\right)-f\left(x_{i,2},w_{0}\right)∥}_{2}^{2},$$

where $x_{i,1}$, $x_{i,2}$ stand for input pair-data to update the Siamese network. The parameters in $w_{0}$ belong to layers FC1 and FC2, and $f(\cdot ,w_{0})$ is the embedding function referred to the Siamese network. The $w_{0}$ is updated using an online dataset during the tracking process. In the rest of this paper, we use $f(x_{i})$ in place of $f(x_{i},w_{0})$ for simplicity.

For online model updating, we collect tracking results and sample extra candidates to maintain an online training set. During the tracking process, we only keep *Q* groups’ tracking results before the current frame (i.e., when the current frame number P<Q), only *P* groups’ tracking results are saved.). In addition, the data from the first frame is always kept. With the online updating, compared to SINT and SiamFC, the proposed Siamese network can learn from the domain knowledge and adapt to appearance changes of specific tracking targets.

### 3.2. Loss Function

#### 3.2.1. Cross-Entropy Loss

The cross-entropy loss [21] is commonly used in the classification task. In the visual tracking task, there are two classes: target and background. The corresponding cross-entropy loss is

(10) $$L_{cls}=\frac{1}{2M}\sum_{i=1}^{M}\sum_{j=1}^{2}-y_{i,j}\log\left(p\left(f\left(x_{i,j}\right),w_{1}\right)\right)-\left(1-y_{i,j}\right)\log\left(p\left(f\left(x_{i,j}\right),w_{2}\right)\right),$$

where

(11) $$p\left(f\left(x_{i,j}\right),w_{1}\right)+p\left(f\left(x_{i,j}\right),w_{2}\right)=1.$$

In addition, $p(f\left(x_{i,j}\right),w_{1})$ denotes the probability that the candidate $x_{i,j}$ belongs to the target. The variable $y_{i,j}$ stands for the true class label as depicted in Figure 1. The parameters in $w_{1},w_{2}$ are from layer FC3. The batch-size of input pair-data is *M*. Under the cross-entropy loss, the classification model attempts to approximate the true distribution of candidate. The classification model based on the cross-entropy loss mainly focuses on increasing the margin among candidates from different classes. As discussed in Section 1, we also want to decrease the distance among feature vectors, which are extracted from the same target. However, the cross-entropy loss does not have restriction about the intra-class variations, which is essential to the visual tracking task.

#### 3.2.2. Contrastive Loss

In the metric-based learning, the contrastive loss [19] is mainly used to reduce the intra-class variations by pulling feature vectors from the same instance together, and it is given by

(12) $$L_{con}=\frac{1}{2M}\sum_{i=1}^{M}s_{i}∥f\left(x_{i,1}\right)-f\left(x_{i,2}\right){∥}_{2}^{2}+\left(1-s_{i}\right)\max(0,m-∥f\left(x_{i,1}\right)-f\left(x_{i,2}\right){∥}_{2}^{2}).$$

The variable of *m* is a hyperparameter. As shown in Figure 1, the variable *s* stands for the pair label. If two candidate samples $\left(x_{i,1},x_{i,2}\right)$ are from the same object (target or background), the value of variable $s_{i}$ is 1. Otherwise, it is 0. It is not easy to train the Siamese network supervised by the contrastive loss. For example, if we have $N_{1}$ target candidates and $N_{2}$ background candidates, the possible input sample pairs are $N_{1}\left(N_{1}-1\right)/2+N_{2}\left(N_{2}-1\right)/2+N_{1}N_{2}$. It would be computationally prohibitive to use all possible sample pairs to update the Siamese network. In addition, although the contrastive loss tends to increase the inter-class variations, the obtained margin may not be satisfactory for the discrimination purpose.

#### 3.2.3. Improved Contrastive Loss

In practice, the contrastive loss employed a function with a hard decision margin to separate object $f\left(x_{i,1}\right)$ from background $f(x_{i,2})$:

(13) $$E=\max(0,m-∥f\left(x_{i,1}\right)-f\left(x_{i,2}\right){∥}_{2}^{2}).$$

The partial derivative of *E* on *f* is zero in the range of (m,∞):

(14) $$\begin{array}{c} \frac{\partial E}{\partial f(x_{i,1})}=\left\{\begin{array}{c} -2\left(f\left(x_{i,1}\right)-f\left(x_{i,2}\right)\right),\;{∥f\left(x_{i,1}\right)-f\left(x_{i,2}\right)∥}_{2}^{2}\leq m,\\ 0,\;other, \end{array}\right. \end{array}$$

(15) $$\begin{array}{c} \frac{\partial E}{\partial f(x_{i,2})}=\left\{\begin{array}{c} -2\left(f\left(x_{i,2}\right)-f\left(x_{i,1}\right)\right),\;{∥f\left(x_{i,1}\right)-f\left(x_{i,2}\right)∥}_{2}^{2}\leq m,\\ 0,\;other. \end{array}\right. \end{array}$$

However, the Softmax loss behaves as a smooth function to separate object $f\left(x_{i,1}\right)$ from background $f(x_{i,2})$:

(16) $$E=-log\left(\frac{e^{f{\left(x_{i,1}\right)}^{T}w_{1}}}{e^{f{\left(x_{i,1}\right)}^{T}w_{1}}+e^{f{\left(x_{i,1}\right)}^{T}w_{2}}}\right)-log\left(\frac{e^{f{\left(x_{i},2\right)}^{T}w_{2}}}{e^{f{\left(x_{i,2}\right)}^{T}w_{1}}+e^{f{\left(x_{i,2}\right)}^{T}w_{2}}}\right).$$

The weights of $w_{1}$ and $w_{2}$ correspond to object and background, respectively. In addition, the gradients to separate the object from background will persist:

(17) $$\frac{\partial E}{\partial f(x_{i,1})}=-\left(1+\frac{e^{f{\left(x_{i,1}\right)}^{T}w_{1}}}{e^{f{\left(x_{i,1}\right)}^{T}w_{1}}+e^{f{\left(x_{i,1}\right)}^{T}w_{2}}}\right)w_{1},$$

(18) $$\frac{\partial E}{\partial f(x_{i,2})}=-\left(1+\frac{e^{f{\left(x_{i,2}\right)}^{T}w_{2}}}{e^{f{\left(x_{i,2}\right)}^{T}w_{1}}+e^{f{\left(x_{i,2}\right)}^{T}w_{2}}}\right)w_{2}.$$

The fixed margin used in the contrastive loss to separate feature vectors can’t handle challenge video sequences very well. Thus, it is not appropriate to separate the object and background only supervised by the contrastive loss. The cross-entropy loss is more suitable to increases the inter-class variations than the contrastive loss. However, the contrastive loss is good at reducing the intra-class variations. Given that, we think these two loss functions are complementary. Our proposed loss function not only increases the inter-class variations but also reduces the intra-class variations via

(19) $$L=L_{cls}+\beta L_{con}+\frac{\lambda }{2M}\sum_{k=0}^{2}{∥w_{k}∥}_{2}^{2}.$$

The last item on the right-side of Equation (19) is the regularization term.

### 3.3. Implementation of the Bayesian Verification Model

For the implementation of the Bayesian verification model, features extracted by the Siamese network are used to update it. In the first frame of a video sequence, the proposed algorithm samples 500 positive candidates and 1000 negative candidates, respectively. Then, the Bayesian verification model will be trained by the EM-like algorithm, which is the same as [22]. In addition, the Bayesian verification model is updated frame by frame. Furthermore, it is adjusted with ten iterations in every frame. In the phase of inference, we utilize Equation (3) to compare two Siamese feature vectors extracted from an image pair and get their similarity scores to select the most relevant candidate bounding boxes. In practice, the top five results will be saved.

## 4. Implementation Details

To improve the performance under scale variation, the strategy of bounding box regression [6] is employed. The bounding box regression model is only initialized with the target state in the first frame. Then, it is used to refine the returned results from random sample candidates. For the online updating, the learning rate is η=0.005 while the number of iterations is T=50 in the first frame. The batch size of the input data is M=256. In the subsequent frames, the Siamese network is adjusted with a learning rate of η=0.003 and T=30 iterations. The hyperparameters of *m* and β in the loss function (19) are both set to 1. Our proposed Siamese network is adjusted every six frames in order to prevent overfitting with the recent training samples. As for the joint Bayesian verification model, it is iterated 50 times in the first frame and 10 times in subsequent frames. The Siamese network is updated by stochastic gradient descent (SGD). The joint Bayesian verification model is updated frame by frame.

## 5. Experimental Validations

In this section, the proposed algorithm (OSNV) is evaluated on three large benchmarks, OTB-2013 [14], OTB-50 and OTB-2015 [29]. They contain 51, 50 and 100 test sequences, respectively. In addition, we test our proposed algorithm in VOT-2016 [17] and TempleColor [31]. Our proposed algorithm is implemented in MATLAB 2017a on a Dell R7300 desktop with a Nvidia TitanX GPU. The algorithm runs at 1.2405 fps on average.

The tracking algorithms are evaluated based on the distance precision and bounding box overlap with one-pass evaluation (OPE), temporal robustness evaluation (TRE) and spatial robustness evaluation (SRE). The predicted rectangle boxes are considered to be a success if their coordinate centers are less than 20 pixels compared to the ground truth bounding boxes. The numbers in the legends of the distance precision plots are the precision value when the location error threshold is 20 pixels. For the success rate plots, they stand for the area under curve. When the overlap value is more than the overlap threshold, it is a success prediction.

### 5.1. Ablation Study

To evaluate the performance of the proposed improved contrastive loss and Siamese network, four additional algorithms are designed: (1) The algorithm OSNV_Log is the proposed Siamese network with the logistic loss. For the ablation algorithm OSNV_Log, the layer contrastive loss is disabled with the replacement by the logistic loss layer, as shown in Figure 1. In addition, the layer of FC3 and cross entropy (Softmax) layer are also disabled. (2) The algorithm OSNV_Sof is the proposed Siamese network with the cross-entropy loss. This is implemented by disabling the layer L2D and contrastive loss in Figure 1. (3) The algorithm OSNV_Con is the proposed Siamese network with the contrastive loss, which is implemented by disabling the layer FC3 and cross-entropy loss in Figure 1; (4) For the algorithm OSNV_PCA, the proposed Siamese network is disabled. The PCA is applied to reduce the dimension of VGG-M outputs from 3×3×512×1 to a 256×1 vector, and its length is the same as the output of the proposed Siamese network.

**Figure 3:**
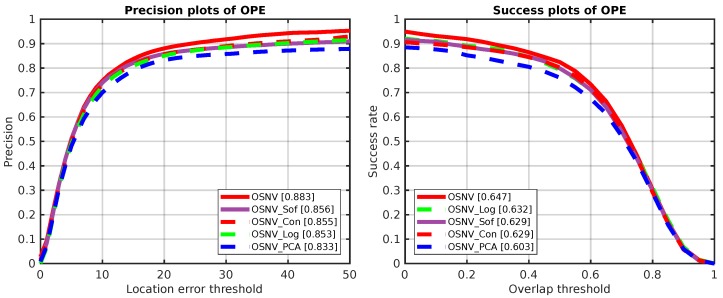
The ablation study results of OSNV on OTB-2013 with extra four algorithms. The plot on the **left side** is the precision amplitude varied with location error threshold, and the legend is about precision scores. The plot on the **right side** is the success plots of OPE on OTB-2013, best viewed in colour.

The ablation study results are depicted in Figure 3. Compared with the contrastive loss or cross-entropy loss, the proposed improved contrastive loss gains improvement about 1.8% on the success rate. It can also noticed that the method with logistic loss is better than the cross-entropy loss or contrastive loss. Our proposed improved contrastive loss (OSNV) outperforms the logistic loss (OSNV_Log) by 1.5%. Furthermore, when the input data is only adjusted by the PCA model, the performance has a big degradation about 4.4% compared to the OSNV. The performance degradation is attributed to discarding domain knowledge and not adapting to appearance changes of target. Basically, based on the ablation results, it can conclude that the contrastive loss and cross-entropy loss compensate for each other. More importantly, the ablation results have opened an interesting topic: Are the loss functions used in current Siamese-like algorithms suitable for model training regardless of the structures of neural networks?

### 5.2. Evaluation on OTB-2013

**Quantitative Evaluation**

To illustrate the characteristics of our proposed algorithm, we compare the OSNV algorithm with nine state-of-the-art tracking methods. According to their working principles, these algorithms could be classified into four classes: (i) Siamese-like tracking algorithms, including SiamFC_3s [7], and SINT_noflow [9]. Both of them train an offline Siamese network to extract feature vectors. (ii) algorithms based on CNNs: MDNet [10], SANet [11]; (iii) algorithms based on correlation filter e.g., ECO [12], KCF [32], MCPF [13]; (iv) algorithms based on hand-crafted features e.g., MEEM [33], TGPR [34].

**Figure 4:**
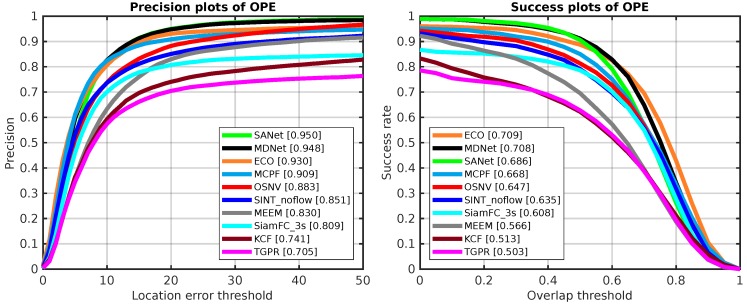
The OPE results of 10 tracking algorithms on OTB-2013. The **left side** is precision plots and the **right side** is success plots, which are both evaluated on OTB-2013.

The overall performance of all algorithms are displayed in Figure 4. The plot on the left side is the location error threshold with OPE. The plot on the right side is the success rate with OPE. There are 51 video sequences to be tested with the evaluation tool provided by OTB-2013 [14].

As shown in Figure 4, our proposed algorithm OSNV achieves a comparable result. Compared with the Siamese-like tracking algorithms: SiamFC_3s and SINT_noflow, the OSNV makes a performance gain about 1.2% and 3.9% on the success rate of OTB-2013, respectively. In addition, the OSNV performs not as good as the state-of-the-art CNN algorithms MDNet [10] and SANet [13]. There are two reasons: (1) the training data used by MDNet and SANet comes from the VOT dataset, which is more appropriate for tracking task. However, the data used in MDNet and SANet for backbone network training is not completely independent to test video sequences, which is not supported by the VOT-Challenge office [17]. (2) these two algorithms apply hard example mining strategy for model updating, which is not used in OSNV. As the correlation filter algorithm ECO [12], it extracts feature vectors by CNNs, Colormap, and histogram of oriented gradient (HOG). And its feature diversity is superior to OSNV. For the MCPF [13], the author combines the particle filter and correlation filter algorithms to improve the tracker’s performance. However, the OSNV takes a random sample strategy, which is not effective as the particle filter.

**Robustness Evaluation**

To evaluate the algorithms’ robustness, Wu et al. [14] introduced two new metrics: TRE (i.e., tracking starts at different frames) and SRE (i.e., some of tiny disturbance is added into the initial state of bounding box in the first frame: $x+\varepsilon_{1},y+\varepsilon_{2},w+\varepsilon_{3},h+\varepsilon_{4}$). For a test video sequence, TRE would generate 20 groups of test sequences about the original one with different start points. SRE is about evaluating a tracking algorithm with 12 different shifted bounding boxes in the first frame based on one annotated video sequence. With the limited GPU cards, we didn’t make TRE and SRE experiments of SANet. In our machine, the tracking speed of SANet is no more than 0.00073 fps.

**Figure 5:**
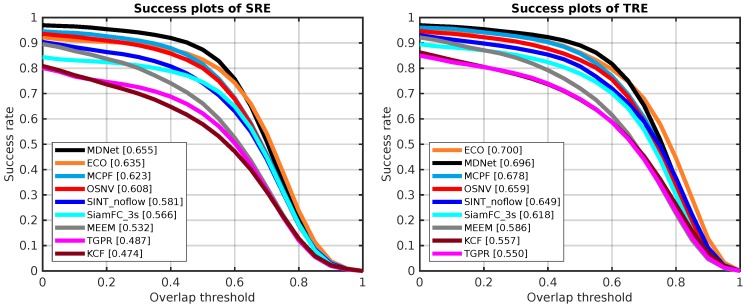
The robustness evaluation results of nine tracking algorithms on OTB-2013. The **left side** is the success plots of spatial robustness evaluation (SRE). In addition, the **right side** is the success plots of temporal robustness evaluation (TRE).

As Figure 5 depicted, the OSNV gets the fourth place in both temporal and spatial variations. For the evaluation metric SRE, the OSNV gains improvement about 4.2% compared with SiamFC_3s. In addition, the OSNV outperforms the algorithm of SINT_noflow about 1% with the mean success rate on the bounding box overlap in TRE. Due to the online model updating, our algorithm has made a good performance in TRE and SRE compared to SiamFC_3s and SINT_noflow.

**Attribute-Based Evaluation**

**Figure 6:**
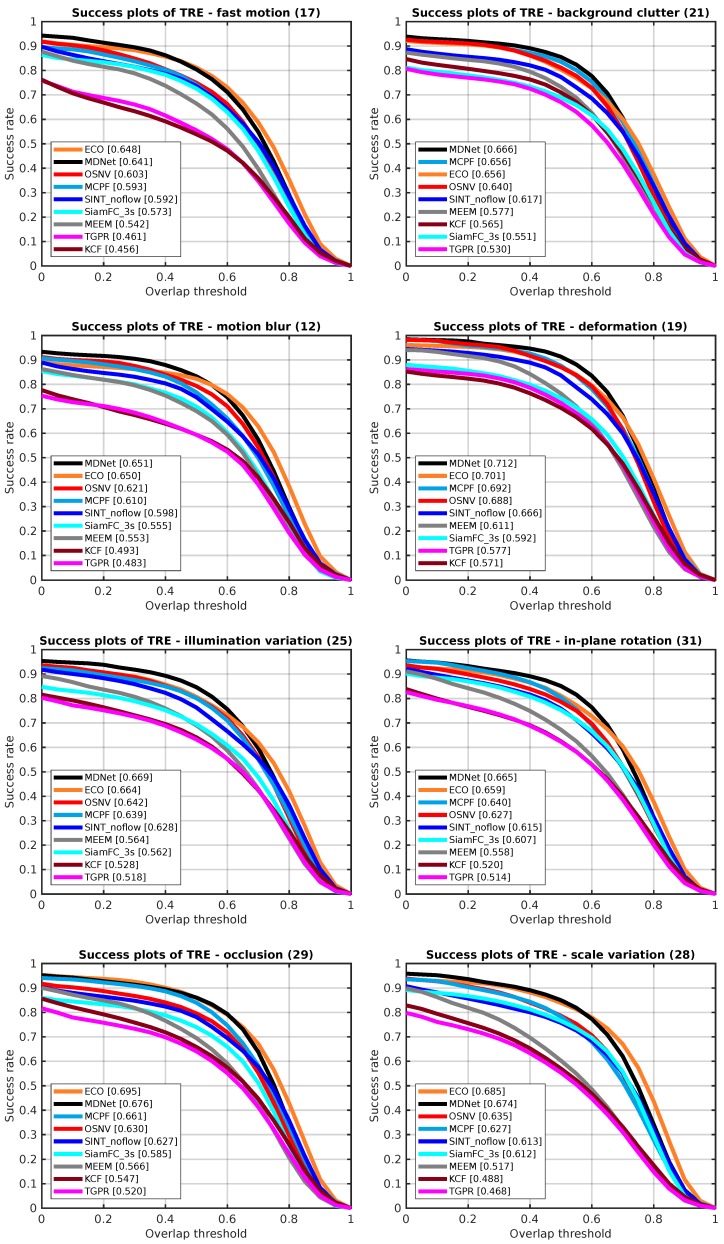
The attribute-based evaluation results of TRE with nine tracking algorithms on OTB-2013.

In [14], Wu et al. proposed to categorize the sequences by annotating them with 11 attributes. In this paper, we show comparison results on eight attributes: fast motion, background clutter, motion blur, deformation, illumination variation, in-plane rotation, occlusion, and scale variation. The success plots of TRE with nine tracking algorithms on different attributes are depicted in Figure 6. The evaluation metric is the success rate with overlap threshold on TRE. The number in every sub figure’s title indicates the amount of video sequences belongs to that attribute. The performance of algorithms corresponding to those eight different attributes represents the ability of the tracking method to deal with different challenges.

The attributes of fast motion, background clutter, motion blur, deformation, in-plane rotation, and illumination are mainly relative to the feature model’s representation ability. As Figure 6 depicted, our proposed algorithm outperforms SiamFC_3s and SINT_noflow on all attributes. For the illumination and scale variation, compared to MCPF, the OSNV makes gains about 0.9% and 0.8%, respectively.

### 5.3. Evaluation on OTB-2015

**Figure 7:**
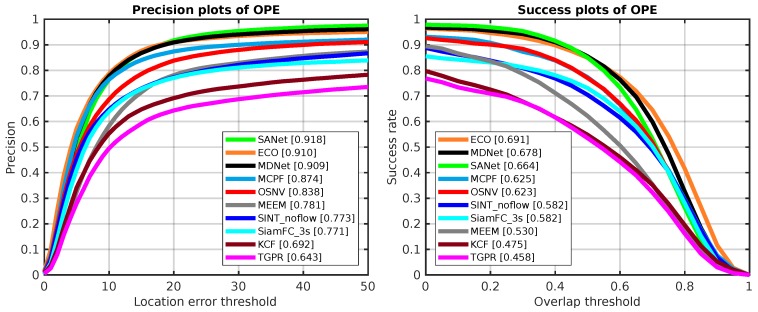
The OPE results of 10 tracking algorithms on OTB-2015. The **left side** is precision plots and the **right side** is success plots, which are both evaluated on OTB-2015.

Here, we compare the proposed algorithm OSNV with nine state-of-the-art tracking algorithms on the challenge tracking benchmark OTB-2015 [29], which has 100 test video sequences, and the size of it is twice as big as OTB-2013. As shown in Figure 7, the proposed algorithm OSNV achieves a comparable result against MCPF on success plots. For the algorithm SiamFC_3s and SINT_noflow, the OSNV has demonstrated superior performance in two metrics.

### 5.4. Evaluation on OTB-50

**Figure 8:**
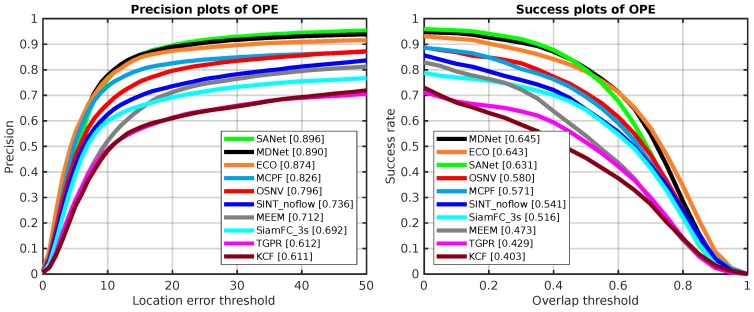
The OPE results of 10 tracking algorithms on OTB-50. The **left side** is precision plots and the **right side** is success plots, which are both evaluated on OTB-50.

As for the dataset OTB-50, it has 50 video sequences which are selected from the OTB-2015. They are more challenging compared to other 50 video sequences within the OTB-2015. Similarly, we evaluate the 10 algorithms on OTB-50 as well. And the test results are depicted as Figure 8. Compared with MCPF, SiamFC_3s, and SINT_noflow, our proposed algorithm makes a consistent improvement.

### 5.5. Evaluation on VOT-2016

For the performance comparison in VOT-2016, the latest Visual Object Tracking toolkit is used. We download the performance results of trackers CCOT [35], MDNet [10], DeepSRDCF [36], SRDCF [27], TGPR [34], and HCF [37] from the VOT-2016 challenge results link. The HCF is an improved version of CF2 [8] with scale estimation. The result of SiamFC_3s is evaluated by our own through adding a vot wrapper for the source code. For the algorithm of SINT, we didn’t use its result for comparison for the reason that its training data is officially not supported by the VOT-Challenge. The overall comparison results are shown in Table 1. Our proposed algorithm gets the second best performance in all three metrics among 8 trackers. Specifically, compared to MDNet, the OSNV has made a gain about 5.46% in EAO. As suggested by VOT office [17], the training data used by MDNet is coming from Imagenet [16], which is different from the original source code.

### 5.6. Evaluation on TempleColor

**Figure 9:**
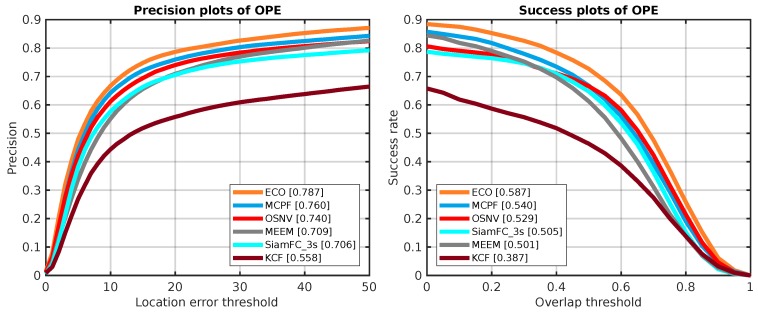
The OPE results of six tracking algorithms on TempleColor. The **left side** is precision plots and the **right side** is success plots, which are both evaluated on TempleColor.

The dataset of TempleColor has 129 video sequences. With the source codes of ECO, MDNet, SANet, MCPF, SiamFC_3s, SINT_noflow, KCF, MEEM, and TGPR downloading from authors’ project pages, we use the OTB [29] evaluation tool to generate the OPE comparison results in TempleColor. For the tracker TGPR, it has a bug to test the video sequence Pool_ce3 in TempleColor. As for the MDNet and SANet, they also have bugs in video sequence Pool_ce1 and Table_tennis_ce, respectively. Thus, we exclude all of them for comparison. In addition, the training dataset of SINT is not independent from TempleColor. Thus, we also exclude it. The performance results are depicted in Figure 9. Our proposed algorithm makes a gain about 2.4% compared with the SiamFC_3s.

### 5.7. Qualitative Evaluation

**Figure 10:**
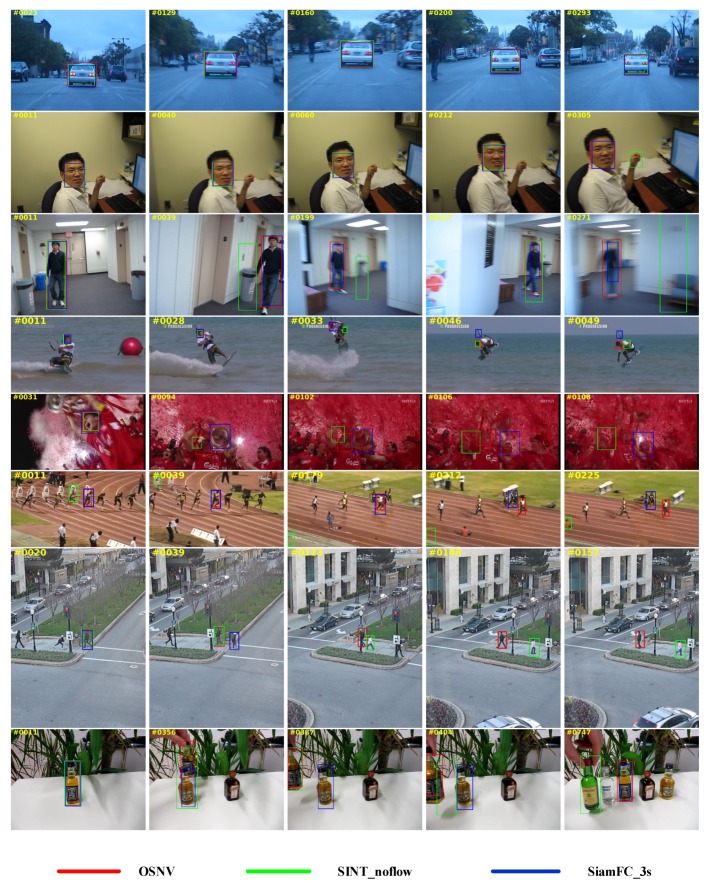
Qualitative performance of our proposed algorithm (OSNV), SINT_noflow, SiamFC_3s on eight challenging video sequences (from top to bottom rows are BlurCar, BlurFace, BlurBody, KiteSurf, Soccer, Bolt2, Human3a and Liquor.)

We select two representative tracking algorithms to compare with the OSNV to perform qualitative results on eight challenging sequences. For each video sequence, we select five images to be shown. The performance results are shown in Figure 10.

In the video sequence of BlurCar and BlurFace, the appearance of both targets doesn’t change much. In addition, the algorithms of OSNV, SINT_nolfow, and SiamFC_3s could accurately estimate the trajectory of targets. However, when targets have undergone dramatic appearance changes as shown in the video sequences of BlurBody, KiteSurf, and Soccer, the performance of SINT_noflow and SiamFC_3s has big degradation. As for our proposed algorithm OSNV, it can adapt to the appearance changes of targets with online updating. In addition, from the video sequences of Bolt2, Human3, and Liquor, we can draw a conclusion that the offline trained Siamese models of SINT and SiamFC are easily disturbed by the similar objects, while the OSNV can predict the location of targets perfectly. We attribute that the OSNV can learn from the domain knowledge from the specific tracking target.

### 5.8. Failure Case

**Figure 11:**
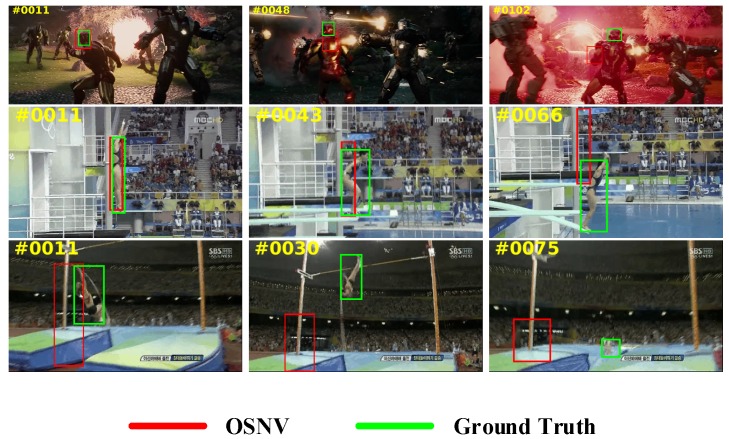
Failure cases of OSNV algorithm about three video sequences: Diving, Ironman, Jump, from top to bottom.

In practice, we find that the OSNV doesn’t perform well in the severe deformation scenes. In Figure 11, we select three frames in each of the three video sequences: Diving, Ironman, and Jump. When the targets undergo severe deformation as Ironman, the OSNV lost the targets immediately. In addition, because of the online updating, the Siamese network has learned lots of incorrect knowledge. Therefore, the algorithm cannot recover from the mistake. Similarly, for the dramatic scale changes within targets, the OSNV cannot deal with that as well. In video sequences of Diving and Jump, our proposed algorithm is unable to accurately predict position and scale changes.

## 6. Conclusions

In this paper, an online Siamese network and improved loss function have been introduced for visual object tracking. Compared with other offline-trained Siamese-like tracking algorithms, the new technique can learn from domain knowledge and adapt to appearance changes of the target. The improved loss function makes a significant improvement over the logistic loss, cross-entropy loss, and contrastive loss. Numerical results indicated that our proposed algorithm outperforms the offline-trained Siamese-like algorithms. However, compared to state-of-the-art trackers MDNet, SANet, and ECO, our proposed algorithm does not perform well in the case of severe deformation, which could be solved by designing a more complex Siamese network in future. In addition, the Bayesian verification model is used for candidate selection, which is different from other tracking methods. Finally, we think that there are two prospects with Siamese network for the future: (1) the offline Siamese-like algorithm with the online model updating may be a promising direction for the promotion of tracking performance; (2) the loss functions used in current Siamese-like algorithms are not suitable for model training regardless of the structure of neural networks.

## Figures and Tables

**Table 1:** The baseline results in VOT-2016. The red fonts with under line, blue bold fonts and green italic fonts indicate the best, the second best and the third best performance. The EAO means expected average overlap, best viewed in color.

	CCOT	SiamFC_3s	DeepSRDCF	SRDCF	MDNet	TGPR	HCF	OSNV
**Overlap**	*0.5332*	0.5081	0.5231	0.5285	0.5366	0.4517	0.4372	**0.5345**
**Failures**	16.5817	32.3730	*20.3462*	28.3167	21.0817	41.0121	23.8569	**17.5017**
**EAO**	0.3310	0.2300	*0.2763*	0.2471	0.2572	0.1811	0.2203	**0.3309**

## References

[1] Benfold B., Reid I. Stable Multi-Target Tracking in Real-Time Surveillance Video. Proceedings of the IEEE Conference on Computer Vision and Pattern Recognition (CVPR 2011).

[2] Chen P., Dang Y., Liang R., Zhu W., He X. (2018). Real-time object tracking on a drone with multi-inertial sensing data. IEEE Trans. Intell. Transp. Syst..

[3] Rautaray S.S., Agrawal A. (2015). Vision based hand gesture recognition for human computer interaction: A survey. Artif. Intell. Rev..

[4] Krizhevsky A., Sutskever I., Hinton G.E. Imagenet classification with deep convolutional neural networks. Proceedings of the Advances in Neural Information Processing Systems (NIPS 2012).

[5] Shelhamer E., Long J., Darrell T. (2017). Fully convolutional networks for semantic segmentation. IEEE Trans. Pattern Anal. Mach. Intell..

[6] Ren S., He K., Girshick R., Sun J. (2017). Faster R-CNN: Towards Real-Time Object Detection with Region Proposal Networks. IEEE Trans. Pattern Anal. Mach. Intell..

[7] Bertinetto L., Valmadre J., Henriques J.F., Vedaldi A., Torr P.H.S. Fully-convolutional siamese networks for object tracking. Proceedings of the European Conference on Computer Vision Workshops (ECCV 2016).

[8] Ma C., Huang J.B., Yang X., Yang M.H. Hierarchical convolutional features for visual tracking. Proceedings of the IEEE International Conference on Computer Vision (ICCV 2015).

[9] Tao R., Gavves E., Smeulders A.W.M. Siamese instance search for tracking. Proceedings of the IEEE Conference on Computer Vision and Pattern Recognition (CVPR 2016).

[10] Nam H., Han B. Learning Multi-domain Convolutional Neural Networks for Visual Tracking. Proceedings of the IEEE Conference on Computer Vision and Pattern Recognition (CVPR 2016).

[11] Fan H., Ling H. SANet: Structure-Aware Network for Visual Tracking. Proceedings of the IEEE Conference on Computer Vision and Pattern Recognition Workshops (CVPRW 2017).

[12] Danelljan M., Bhat G., Khan F.S., Felsberg M. ECO: Efficient Convolution Operators for Tracking. Proceedings of the IEEE Conference on Computer Vision and Pattern Recognition (CVPR 2017).

[13] Zhang T.Z., Xu C.S., Yang M.H. Multi-task Correlation Particle Filter for Robust Object Tracking. Proceedings of the IEEE Conference on Computer Vision and Pattern Recognition (CVPR 2017).

[14] Wu Y., Lim J., Yang M.H. Online object tracking: A benchmark. Proceedings of the IEEE Conference on Computer Vision and Pattern Recognition (CVPR 2013).

[15] Dalal N., Triggs B. Histograms of oriented gradients for human detection. Proceedings of the IEEE Conference on Computer Vision and Pattern Recognition (CVPR 2005).

[16] Russakovsky O., Deng J., Su H., Krause J., Satheesh S., Ma S., Huang Z., Karpathy A., Khosla A., Bernstein M. (2015). Imagenet large scale visual recognition challenge. Int. J. Comput. Vis..

[17] Kristan M., Leonardis A., Matas J., Felsberg M., Pflugfelder R., Čehovin L., Vojír T., Häger G., Lukežič A., Fernández G. The Visual Object Tracking VOT2016 Challenge Results. Proceedings of the European Conference on Computer Vision Workshps (ECCV 2016).

[18] Chatfield K., Simonyan K., Vedaldi A., Zisserman A. Return of the Devil in the Details: Delving Deep into Convolutional Nets. Proceedings of the British Machine Vision Conference (BMVC 2014).

[19] Hadsell R., Chopra S., LeCun Y. Dimensionality reduction by learning an invariant mapping. Proceedings of the IEEE Conference on Computer Vision and Pattern Recognition (CVPR 2006).

[20] Wang N.Y., Yeung D.Y. Learning a deep compact image representation for visual tracking. Proceedings of the Advances in Neural Information Processing Systems (NIPS 2013).

[21] De Boer P., Kroese D., Mannor S., Rubinstein R. (2005). A tutorial on the cross-entropy method. Ann. Oper. Res..

[22] Chen D., Cao X., Wang L., Wen F., Sun J. Bayesian face revisited: A joint formulation. Proceedings of the European Conference on Computer Vision (ECCV 2012).

[23] Sun Y., Chen Y., Wang X., Tang X. Deep learning face representation by joint identification-verification. Proceedings of the Advances in Neural Information Processing Systems (NIPS 2014).

[24] Girshick R., Donahue J., Darrell T., Malik J. (2016). Region-Based Convolutional Networks for Accurate Object Detection and Segmentation. IEEE Trans. Pattern Anal. Mach. Intell..

[25] Guo Q., Feng W., Zhou C., Huang R., Wan L., Wang S. Learning Dynamic Siamese Network for Visual Object Tracking. Proceedings of the IEEE International Conference on Computer Vision (ICCV 2017).

[26] Wang Q., Gao J., Xing J., Zhang M., Hu W. (2017). DCFNet: Discriminant Correlation Filters Network for Visual Tracking. arXiv.

[27] Danelljan M., Hager G., Shahbaz Khan F., Felsberg M. Learning Spatially Regularized Correlation Filters for Visual Tracking. Proceedings of the IEEE International Conference on Computer Vision (ICCV 2015).

[28] Huang G.B., Ramesh M., Berg T., Learned-Miller E. (2014). Labeled Faces in the Wild: Updates and New Reporting Procedures.

[29] Wu Y., Lim J., Yang M.H. (2015). Object tracking benchmark. IEEE Trans. Pattern Anal. Mach. Intell..

[30] Hinton G.E., Srivastava N., Krizhevsky A., Sutskever I., Salakhutdinov R.R. (2012). Improving neural networks by preventing co-adaptation of feature detectors. arXiv.

[31] Liang P., Blasch E., Ling H. (2015). Encoding Color Information for Visual Tracking: Algorithms and Benchmark. IEEE Trans. Image Process..

[32] Henriques J.F., Caseiro R., Martins P., Batista J. (2015). High-Speed Tracking with Kernelized Correlation Filters. IEEE Trans. Pattern Anal. Mach. Intell..

[33] Zhang J., Ma S., Sclaroff S. MEEM: Robust Tracking via Multiple Experts using Entropy Minimization. Proceedings of the European Conference on Computer Vision (ECCV 2014).

[34] Gao J., Ling H., Hu W., Xing J. Transfer Learning Based Visual Tracking with Gaussian Processes Regression. Proceedings of the European Conference on Computer Vision (ECCV 2014).

[35] Danelljan M., Robinson A., Khan F., Felsberg M. Beyond Correlation Filters: Learning Continuous Convolution Operators for Visual Tracking. Proceedings of the European Conference on Computer Vision (ECCV 2016).

[36] Danelljan M., Häger G., Khan F.S., Felsberg M. Convolutional Features for Correlation Filter Based Visual Tracking. Proceedings of the IEEE Conference on International Conference on Computer Vision Workshops (ICCV 2015).

[37] Ma C., Huang J.B., Yang X., Yang M.H. (2018). Robust Visual Tracking via Hierarchical Convolutional Features. IEEE Trans. Pattern Anal. Mach. Intell..

